# HIV testing and counselling experiences: a qualitative study of older adults living with HIV in western Kenya

**DOI:** 10.1186/s12877-018-0941-x

**Published:** 2018-10-25

**Authors:** Jepchirchir Kiplagat, Susann Huschke

**Affiliations:** 1 Moi University, College of Health Sciences, School of Medicine, Nandi Road, Eldoret, Kenya; 2 Academic Model Providing Access to Healthcare (AMPATH) Program, Moi Teaching and Referral Hospital Campus, Nandi Road, Eldoret, Kenya; 3 The University of Witwatersrand, School of Public Health, 27 St Andrews Road, Parktown, Johannesburg, 2193 South Africa; 4 Graduate Entry Medical School, University of Limerick, Castletroy, Limerick, V94 T9PX Ireland; 5 School of History, Anthropology, Philosophy and Politics, Queen’s University Belfast, 25 University Square, Belfast, BT7 1NN UK

**Keywords:** Older adults, Kenya, HIV testing, Ageism, Subjective experiences

## Abstract

**Background:**

Finding HIV infected persons and engaging them in care is crucial in achieving UNAIDS 90–90-90 targets; diagnosing 90% of those infected with HIV, initiating 90% of the diagnosed on ART and achieving viral suppression in 90% of those on ART. To achieve the first target, no person should be left behind in their access to HIV testing services. In Kenya, HIV prevention and testing services give less emphasis on older adults. This article describes HIV testing experiences of older adults living with HIV and how their age shaped their interaction and treatment received during HIV testing and diagnosis.

**Methods:**

We conducted a qualitative study in two HIV clinics (rural and urban) in western Kenya, and recruited 57 HIV infected persons aged ≥50 years. We conducted in depth interviews (IDIs) with 25 participants and 4 focus group discussions (FGDs) with a total of 32 participants and audio recorded all the sessions. Participants recruited were aged between 54 and 79 years with 43% being females. We transcribed audio records and analyzed the data using thematic content analysis method.

**Results:**

Older persons’ experiences with HIV testing depended on where they tested (hospital or community setting); whether they actively sought the testing or not; and the age and gender of the healthcare provider who conducted the test. Participants expressed concerns with ageist discrimination when actively seeking HIV care testing services in hospital settings, characterized by providers’ reluctance or refusal to test. The testing and counseling sessions were described as short and hurried within the hospital settings, whereas the interactions with service providers in home-based testing were experienced as appropriate and supportive. Participants in this study expressed preference for healthcare providers who were older and of similar gender.

**Conclusion:**

HIV testing services are still not tailored to target older adults’ needs in our setting resulting in late diagnosis among older persons. We argue that a scale-up of community level testing services that provide adequate testing and counselling time and actively reach out to older adults is key to attaining the UNAIDS targets of having 90% of PLWH know their status.

**Electronic supplementary material:**

The online version of this article (10.1186/s12877-018-0941-x) contains supplementary material, which is available to authorized users.

## Background

UNAIDS and WHO estimate that over 36.7 million people are currently living with HIV worldwide [1], with more than two thirds of this population residing in sub-Saharan Africa (SSA) [2]. According to UNAIDS [3], Kenya ranks the 4th largest HIV epidemic globally with about 1.6 million people living with HIV and an adult prevalence of 5.4%. Older people, that is ≥50 years, represent a growing share of the HIV population, making up 12% of the total people living with HIV (PLWH) globally [1]. In Kenya, the prevalence among older adults is estimated to be slightly higher than the country’s prevalence – about 5.6%. This number is expected to rise in the coming years due to the wide access of antiretroviral treatment (ART) where people infected with HIV at a younger age are growing older as well as new infections occurring among the older adults.

Among those living with HIV in sub-Saharan Africa approximately 40%, and in Kenya 53%, do not know their status [4]. Though the Kenyan Government has adopted various testing strategies including voluntary counselling and testing (VCT), provider-initiated testing and counselling (PITC), diagnostic testing and counselling (DTC), home-based counselling and testing (HBCT), and recently self-testing, older adults still lag behind in accessing HIV testing services compared to other age groups. There are several reasons for this: first, very little HIV prevention education is targeted at older people [5], second, health care providers may not test older people for HIV [6], and third, older people may lack awareness of the risk factors for getting HIV [7]. As a result, older adults are deprived of the benefits of early diagnosis of HIV infection including timely initiation of HIV treatment.

The literature indicates that adults continue to be sexually active into their old age, including sexual practices often associated with younger people such as inconsistent condom use, sex with multiple sexual partners and casual sex [8–12]. Such sexual behaviors put older adults at risk of acquiring HIV from infected partners or transmitting HIV to an uninfected partner. Despite these risk factors, adults aged 50 years and above are less likely to be tested for HIV than younger adults testing [7, 8, 13]. As a consequence, older adults get diagnosed late into the HIV infection, likely after developing symptoms [14], and only when visiting the hospital for health complications. Despite these specific risks and concerns, there is limited literature that has explored older adults’ experiences with HIV testing services. To address this gap in literature, we draw on qualitative interviews with older HIV-infected adults in western Kenya and analyze their experiences in HIV testing, including explorations on access of testing services and how they perceived their interactions with healthcare providers during testing.

## Methods

### Study setting and participants

The study was conducted at two facilities under the Academic Model providing Access to Healthcare (AMPATH) program [15]. The program provides comprehensive HIV care in 135 Ministry of Health (MOH) facilities spread throughout western part of Kenya and is currently the largest program providing free HIV care and treatment in the country [16].

Participants aged 50 years and above at the time of HIV care enrollment were selected from one urban and one rural facility to allow for a comparison of these settings, with a note that prevalence of HIV in Kenya is higher in urban than in rural settings [17]. Both of the selected sites served a large number of older persons when compared to other facilities.

### Study design

Qualitative research methods were used to generate detailed descriptions of experiences of the participants living with HIV. In-depth interviews (IDI) and focus group discussions were employed in this study. The interviews were conducted in Kiswahili, the Kenyan national language, and lasted between 60 and 90 min each. We interviewed 25 participants; 16 (9 males and 7 females) participants from urban facility and 9 (5 males and 4 females) from rural facility. We also conducted four focus group discussions (FGD) with a total of 32 participants; two FGDs (one for male and one for female participants) were conducted in a rural and another two FGDs (also one for male and one for female participants) in the urban facility. Both IDIs and FGDs were used to triangulate information obtained from the participants.

### Study procedures

#### Research ethics

The study was approved by the University of Witwatersrand Heath Research Ethics Committee (Clearance Certificate No: *M160449*) and the Moi University and Moi Teaching and Referral Hospital Institutional Research Ethics Committee (Formal Approval No. *FAN: IREC 1664*). Permission was granted from the AMPATH research program office to recruit participants from its clinical care sites. Prior to the interview, participants were informed about the nature of the study and the methods including the audio recording of the interviews. Participants were assured that the information obtained was going to be kept anonymous and confidentiality was going to be maintained. Participants were also informed that during reporting of the results of the study, quotes were going to be used with no link to their names. All participants who agreed and gave consent to participate in the study and to be audio recorded were interviewed.

#### Inclusion and exclusion criteria

HIV infected men and women aged ≥50 years at the time of HIV care enrollment and receiving care at AMPATH were targeted. For the purpose of this study a cut off age of 50 years for older persons was adopted from the WHO definition [18] for those infected with HIV.

Participants who were currently in care at the two participating outpatient HIV clinics, had been followed up for at least 1 year, and were aged 50 or older at enrolling into HIV care were included in our study. The eligible participants who did not consent to participation and audio-recording were excluded.

Of the total 65 approached participants who consented to participate in our study, we interviewed 57. The other 8 participants invited for the FGD did not turn up due to other commitments during the interview date.

### Data collection

Two research assistants (RAs), recruited from the staff at the social behavioral department within the AMPATH program, were trained by the first author to conduct the data collection. We purposefully selected older research assistants, one female (52 years) and one male (55 years), and each was responsible for interviewing females and males respectively. The choice of older RAs was informed by references in other studies [19] with older adults. The chosen RAs have an educational background in social science and regularly support researchers at AMPATH by collecting qualitative data. We pilot tested the in-depth interview tool (in English) in a peri-urban HIV care clinic to ensure the clarity of the interview questions. After necessary modifications such as deleting redundant questions and including a number of new questions that arose from the pilot testing, the IDI guide (Additional file 1) was then translated from English to Kiswahili.

For FGDs, we recruited four (2 males and 2 females) research assistants, who were not affiliated with the AMPATH program. The RAs, who were all aged above 45 years, with skills in facilitating FGDs, were referred to this study by researchers at the Department of Anthropology, Moi University. The RAs were trained to use the data collection tool (FGD guide – Additional file 2) by the first author. The FGD guide provided similar questions as those of the IDI’s with probes focusing on identified themes or sub themes from IDI transcripts.

#### Participant identification

During the clinic days, research assistants reviewed the list of persons attending the clinic on that day. The records of persons aged 50 years and above were reviewed to determine the age at first engagement in HIV care. The research assistant would then provide the clinician with the list of eligible participants (clinicians had been informed about the study). The clinician would inform the research assistant as soon as the participant completed the clinic visit. The research assistant would then approach and explain the purpose of the study to the participants. If they agreed to participate, a meeting date and time was scheduled at a venue of participant’s convenience to allow privacy and comfort during the interview. All except one participant asked to do the interview on the same day they were in the clinic and wanted it done at the health facility in which they were seeking care at. Contact details were requested for one participant for the purpose of reminder and communication before the meeting time that was scheduled.

FGD participants were identified during the clinic visit, through an outreach worker stationed at the facility. Outreach workers review files of all new patients attending HIV facility and draw the patient tracer card, including patient information and their place of residence, for future follow up if patients fail to attend clinic visits. In the morning of the clinic day, patient files of the expected patients are pulled out. If a patient was 50 years and above at the time of initial enrolment, the file would be flagged with a yellow sticker. After the clinic visit, the clinician would inform the research assistant who would approach the participant and explain the purpose of the study. The consent process would be done and if the participant agreed to take part, contact information was requested for further communication regarding the date, time and venue of the FGD.

#### Interview process

During the in-depth interviews, the research assistant explained the purpose of the study and obtained written informed consent from the participant. There was a total of eight participants who were unable to read and write and a thumb print indicating their consent was obtained. The research assistant also asked whether the participant needed someone they trust to be present during the interview. None of them indicated the need for a companion. The interviews were conducted in one of the two enclosed research rooms within the clinical care space. The room provided privacy for the participants and a quiet environment for audio-recording. The participants’ demographics were captured and they were asked a question to gauge their HIV knowledge. Participants were then invited to narrate their experiences during HIV testing and counseling.

The FGDs were conducted in a room reserved for research outside of the HIV clinic facilities but within the MTRH campus and in a teaching room at a nearby medical training college in Mosoriot. As ‘neutral’ spaces away from the treatment rooms of the clinic, these spaces provided a convenient environment to discuss health facility related factors that were deemed negative. The FGD room was set with an oval table that provided seats for all participants facing each other, with the facilitators sitting among them. This set up provided an opportunity for each participant to contribute to the discussion. Two RAs participated in each FGD (female RAs conducted the female-only FGD while male RAs conducted male-only FGD). One of the RAs facilitated the discussion while the other took notes. Prior to the interviews, participants were informed of the purpose of the study and oral consent was obtained. All FGDs were conducted in Kiswahili and audio-recorded. The FGDs lasted between 90 and 135 min.

#### Data management and analysis

Data obtained from in-depth interviews and focus group discussions were transcribed verbatim, and translated from Kiswahili to English. All the transcripts were then uploaded to NVIVO version 10 for coding. A pre-developed codebook based on the literature on facilitators and barriers for seeking HIV testing services and accessing care was used during coding. The two RAs who conducted the IDIs and the first author coded the first three transcripts, which allowed the team to revise the codebook based on the analysis of these transcripts. The first author then shared the revised codes with the second author (SH) who made some recommendations to further clarify the structure of the codebook. The final codebook was agreed upon by the authors and the RAs. Thematic content analysis [20] was used to describe emerging patterns from in-depth interviews and FGDs. Recurrent themes identified and patterns established from the two data sources were summarized. Information obtained from the FGDs was used to complement the IDIs and to seek data saturation. Mapping and interpretation was done by searching for associations of concepts and explanations in the data. We summarize the findings according to predeveloped themes and the subthemes that emerged during the coding process.

## Results

### Participants’ characteristics

We interviewed a total of 57 participants (25 in-depth interviews and 32 in focus group discussions) with their ages ranging from 54 to 79 years. Participant characteristics are summarized in Table 1.

**Table 1:** Participant characteristics

Variable	n (%)
Age Category (years)	
50–59	19 (33.3)
60–69	28 (49.2)
70–79	10 (17.5)
Sex	
Female	27 (47.4)
Male	30 (52.6)
Clinic Location	
Urban	31 (54.4)
Rural	26 (45.6)
Marital status	
Married	30 (54.4)
Widowed, separated, divorced	27 (45.6)
Level of Education	
No formal education	24 (42.1)
Formal education – Primary	19 (33.3)
Formal education – Secondary	10 (17.5)
Formal education – Tertiary	4 (7.1)

In the sections below, we describe older adults’ experiences with HIV testing and encounters with healthcare providers and how their age shaped their interaction and the treatment they received during their HIV testing and diagnosis.

### Experiences with HIV testing

We found that older persons’ experiences with HIV testing depended on where they tested (hospital or community setting); whether they actively sought the testing or not. Participants expressed concerns with ageist discrimination when actively seeking HIV care testing services in hospital settings, characterized by providers’ reluctance or refusal to test. The testing and counseling sessions were described as short and hurried within the hospital settings, whereas the interactions with service providers in home-based testing were experienced as appropriate and supportive. Age and gender of the healthcare provider also mattered to older adults in seeking HIV testing services. We did not find significant variations in experiences of older adults in urban and rural facilities hence we jointly describe their experiences.

### Testing during hospital visit or during hospitalization

The majority of the participants indicated visits to the hospital as a main avenue to be tested for HIV. Participants described periods when they were sick and sought out-patient services or when they were admitted in hospital for other health conditions. Within the hospital setting, there are two approaches for HIV testing; i) opt-out HIV testing known as provider-initiated testing and counseling (PITC) and ii) opt-in HIV testing approach known as diagnostic testing and counseling (DTC) implemented as part of disease diagnosis. These two strategies of HIV testing and counseling are implemented in all healthcare facilities providing inpatient and outpatient services in Kenya. Some participants tested at the hospital setting received the HIV positive test results with shock, as the following quote exemplifies:

*“I was sick with TB. I was really coughing and when I came to the hospital, I was admitted. They took an x-ray and they told me I have TB. I was also tested for HIV and that is when I found out that I had this disease [HIV]. It was shocking for me. I could never explain where I got it. It has never occurred to me that I could ever get it. At the time, I had lost so much weight”.*

IDI, female, 67 years, rural

Unlike the participant above, another participant tested in the hospital setting was relieved that she finally knew the condition that she had been suffering from. She had been in and out of hospital without proper diagnosis which had increased her health care seeking costs and progressed the condition. Further probing in the interview revealed that the service providers had missed two important opportunities to test this older person earlier on.

*“I began coughing for some time. I went to the hospital and was given drugs for TB. I took the drugs but nothing changed. I grew so thin. I went back to the hospital because I was not improving. I was asked to take an x-ray and was told that the TB was really bad and had eaten all my chest. I was given more medicines and was told to come back after two weeks. Before the two weeks lapsed, I began to have diarrhea and also started vomiting. My face even started dying from one side [paralysis]. I went back to the hospital and I was admitted. A sister came and counseled me for HIV testing and I agreed to test. I tested positive. At this point I now knew what the problem with my body was.”*

FGD, female, 67 years, urban

### Home based counseling and testing

Bringing HIV testing services to the older people (rather than waiting for people to actively seek out testing) proved to be critical for early diagnosis among those infected. Kenya introduced the door-to-door testing campaign after the AMPATH program showed that this strategy yielded results - with 98% of people tested [21]. During the door-to-door HIV testing, also known as home based counseling and testing (HBCT), a certified counsellor trained for HIV counseling and testing was assigned a catchment area of about 500 households. The counselor resided in the assigned area and provided perpetual testing services to his/her catchment site. The counselor went from house to house with the help of community health volunteers, and provided HIV testing services to all persons aged 13 years and above, including the elderly in the household. For all those that tested positive, the counselor worked with the patients to link them to care near them. He/she went back to the household after 3 month to offer testing services to all those who originally tested negative. Because the counselors resided in the assigned catchment area for more than a year, they spent as much time as possible with the residents in counseling before providing HIV testing services. This likely resulted in trust by the residents and acceptability of the tests.

Some of the participants we interviewed got to know their HIV status during the testing services that were provided at their homes. Participants appreciated HBCT services that were led by the AMPATH program. They perceived them as a welcome attempt to reach older persons who would otherwise not visit the hospital settings where HIV testing services are often provided.

*“They came to my home (…) and I was praying to God not to be positive and when I read the results I was shocked, I didn't know where to start from. I just assumed my husband had infected me or they injected me with a used syringe in the hospital. But at least I got to know [my status] before I became too sick. Can you imagine what would have happened if I was not tested then? I probably would be dead now. Maybe, I would have grown so thin and by the time I got medication it would not work on my body anymore.”*

IDI, female, 69 years, urban

Just like the above mentioned participant, another participant also tested during HBCT and attributes their source of infection to their husband.

*“When my husband died I didn't know what killed him so there was a time health workers were walking door to door, they counselled people before testing them. I was tested and I accepted my status, maybe I was infected by my husband”*

IDI, female, 69 years, urban

Yet another participant, also tested during the door to door testing shared her testing experience. She believed she was HIV negative and agreed to be tested, and was deeply shocked when she received positive test results.

*“My husband was really sick in 2008. He became so sick that he had to be admitted in the hospital. He could not eat because he had wounds in his mouth. He was transferred to the district hospital where he continued with medication. He grew so thin and died after two months. No one informed me about the condition he had. After we buried him, we continued with our normal activities. Then doctors who were going from house to house to do HIV testing came to my house. My children were around and the doctor asked if we could be tested. My son was outside and he was sometimes helping the doctors with testing when they moved from house to house. I agreed to test because I knew I was ‘clean’. I was so sure I did not have HIV. Then after the test the doctor told me the results. I was so scared and did not believe it was true. My world came crushing on me. I looked around and knew I was gone [was going to die].”*

FGD, female, 64 years, rural

### Proactive seeking of HIV test

A small number (*n* = 4) of participants proactively sought out an HIV test because they perceived themselves to be at risk of being infected. HIV risk perceptions were informed by condom bursts during sexual intercourse with partners they suspected could be HIV infected, having multiple partners, having taken care of HIV infected person or having signs and symptoms similar to those of HIV infected persons.

One of the female participants intimated on her engagement with multiple sexual partners and described situation of unprotected sex under the influence of alcohol. She visited a rural health facility to seek HIV testing services and was first rejected because she did not have anyone accompanying her, despite that fact that there are no existing policies in Kenya requiring that during HIV testing, one must be accompanied by another person. Her testing experience with the healthcare provider indicates likely ageism in provision of testing services to older persons:

*“For me, I just went to the hospital myself. I had heard about HIV and wanted to test and get to know my status. When I got to the hospital, I found the nurse. I told her that I had come to be tested and she asked if I had come with someone. I told her that I was alone. She asked me to go home and come back the next day with a companion. I insisted that since I had made the choice to be tested I needed to [do it now]. She agreed to test. She pricked my hand and put blood in a small white stick. I could see blood move through the stick. She said that the test is incorrect and probably it can be done some other time. I told the nurse that (…) I still have a lot of blood in my body and we can do another test. We did another test and it came out positive. The nurse told me that the initial test had turned out positive but she was scared to tell me.”*

IDI, female, 56 years, rural

Other participants proactively sought out an HIV test following the death of a partner or a close relative or after an HIV-positive test result of a partner. Participants felt the urge to test out of curiosity, fear of being infected or fear of dying, too. The experiences with healthcare providers in these cases similarly suggest ageism and discrimination. A male participant who visited a local health facility during the early times of the HIV pandemic, when testing services were mainly provided for pregnant women as part of the national antenatal care policy, described his experience. The interaction with the healthcare provider indicates that the provider was neither used to nor comfortable with testing an older man:

*“After [my wife’s] demise, I became sick (…). I had bad dreams to the extent that I was communicating to the dead, and became very weak that I couldn’t walk; I thought I was dying. I decided to go for a test because in my community people believe in witchcraft so I wanted to confirm so that I could tell them it wasn’t witchcraft. I went home to our nearby Health Center and it took time because the doctor was hesitant to test me until I had to threaten him that I would write a letter stating that in case I die, it is my doctor who has killed me. When he heard so, he asked me whether I had made a decision and I told him I was an adult and I had decided. That I had lost my wife and I wanted to know whether it was due to HIV so it took three hours before he agreed to test me”.*

IDI, male, 65 years, urban

Another participant who had lost a husband to AIDS narrated her long process of seeking care for her ailing husband in several hospitals. He was only tested and diagnosed with HIV at a very late stage and died a few months after his diagnosis. She was never offered an HIV test when taking care of her husband at the hospital. After she buried the husband, she decided to get tested for HIV.

*“Since I had heard that it [HIV] is transmitted sexually, I decided to go back to the hospital to get tested. When my turn came, the sister inside the room asked me if I was ready for the results in the likely event that they turn out positive. I told her any results were ok with me. When the results came out, it was positive. I was not shocked because my husband had died of HIV so I knew I could likely have [it but] of course I was praying to God for the results to come out negative”.*

FGD, female, 69 years, rural

Another female participant who had lost a daughter she had cared for to HIV went to seek an HIV test. She had heard that one could get infected if they cleaned an infected person’s wounds without wearing gloves. Her description suggests ageist discrimination by other patients (young people) and healthcare providers:

*“I came to the hospital and found two people, young ones, I think they were lovers, seated outside the office waiting for their turn. I sat next to them, and they stopped conversing and kept sneaking a look at me. When they went in, I stayed alone. Then another doctor passed by and asked if I was ‘lost’ and needed help. He went ahead to explain that the place was for HIV testing, and I say yes, I was waiting in line. He said ok and left, but I could see the shock in his face. I got tested and found with the virus. It was devastating but God has been with me now for 5 years and I am still strong.”*

IDI, female, 67 years, urban

### Limited time spent with providers

Participants intimated that during the testing in hospital settings, it would be beneficial to spend more time with healthcare providers after testing, and to be able to discuss life after an HIV positive result. Older persons are, however, treated in a hurry during the clinic encounter with the clinician. Participants noted that the clinicians were overwhelmed by the number of patients on the queue, hence spend limited time with patients. This was perceived as an issue particularly by the older adults who may need more time to gain trust, ask questions and feel comfortable to discuss sexual matters.

*“After the doctor counseled me, I thought I was prepared to take any results that came. I think I was convinced that I was not infected. Then during the test the doctor and I were there but we were so silent. When the results became positive, the doctor told me to go to AMPATH and ushered in the next person on the queue.”*

IDI, male, 54 years, urban

Unlike the above mentioned participant, a participant tested at her home expressed satisfaction with the time she spent with the doctor who provided HIV testing services and with the subsequent visits thereafter.

*“She even took time to educate me. I asked a lot of questions and she was patient with me. She answered everything until I said I was ready. She then tested me. After the results were out, I could not remember whether one line was positive or not, she again taught me. Until I could see that my results were positive. I wanted to run outside and scream but she held me and calmed me down, she counselled me again. She stayed in my home until noon [i.e. she spent about 4 hours with the patient] and left but she visited me again the next day.”*

IDI, female, 65 years, urban

### Age and gender matter

The age of a care provider was noted to be an important factor for HIV infected older adults during testing. Discussing sex-related issues with a healthcare provider of the same age as your child or grandchild was viewed as a ‘taboo’. During an FGD, male participants unanimously expressed the need to have a male provider who can discuss male-related sexual issues which were considered ‘private’, including erection difficulties and sexually transmitted diseases among others. A participant shared his satisfaction after being attended to by a male provider:

“*He is male. You know, you can talk comfortably to your fellow male and tell him everything about your private life and he would understand. There are things you cannot share with a female doctor, especially if you have private related stuff.*”

IDI, male, 60 years, urban

Female participants also shared similar views and prefer to be attended to by a female health care provider. Additionally, age was considered important and participants explained that an older provider would be ideal to care for older persons. Some of the participants’ views are exemplified below:

*“I wish women who are older, like us grandmothers, can be given our own doctor who would attend to us. That way, we don’t have to push each other with those aged like our children on the queue”.*

FGD, female, 70 years, rural

*“If also we could be given an older female doctor that understands what women go through, it would be easier to tell them even issues that are private and we would not feel embarrassed.”*

FGD, female, 66 years, urban

*“I would prefer if it was a female doctor and a bit older. You cannot talk to young girls or even male [doctors] about issues down there [issues related to sexuality].”*

IDI, female 54 years, rural

## Discussion

This article highlights the experiences of HIV infected older adults with HIV testing services. The varied testing experiences have implications for how older people subsequently engage in HIV care services. As HIV medication continues to be widely accessible, finding the HIV infected, especially older persons, will require strategies that extend beyond facility-based HIV testing services [21–23]. This is important as countries work towards achieving the UNAIDS strategy of ending HIV transmission in 2030 by targeting to find at least 90% of those who are HIV infected, initiating them on ART and attaining viral load suppression.

Findings of our study demonstrate varied experiences of HIV testing among older adults depending on the setting of HIV test provision - whether in a hospital setting or home setting. The findings revealed that older adults are likely to be tested at the hospital in relation to another illness or when very sick and admitted to the hospital. When they test positive for HIV, older patients were shocked and devastated, likely indicating that older adults perceive themselves not to be at risk of HIV infection. These results support previous research work in similar settings [5, 24, 25] which showed that because HIV prevention messaging targets younger adults and other key populations, older adults continue to lack knowledge about HIV prevention [19]. The few older adults that actively seek HIV testing services do so amidst fear of discrimination.

The concept of ageism, ‘prejudice and discrimination against older people’, has been well described in literature in relation to HIV and co-morbidities with issues that include older age and sexuality [26–28]. Ageism has been associated with problems in access to HIV services, resulting in diagnosis late into HIV infection among older adults [5, 25, 28]. Healthcare providers on the other hand incorrectly assume that older adults are no longer sexually active and are more likely to misdiagnose or not diagnose an HIV infected older person presenting with flu-like symptoms [7, 8], or, as our findings show, with comorbid infections such as TB. Our findings also indicated that older adults viewed VCT services to be discriminating against older persons. The signage and directions to the VCT service station were often written in English, which older adults might not speak, or staffed with young providers who are unwilling or uncomfortable in providing services to older persons. HIV testing services were seen to target women, but focused on women at the reproductive age group (15–49 years). Once a woman is no longer in the reproductive age group, they seemed to no longer count [29]. These negative attitudes result in a lack of investments in HIV prevention education, testing and HIV care responses specifically targeting older adults. HIV policies on testing and prevention services in Kenya, and likely most parts of sub-Saharan Africa, should target older adults as a special group at risk of HIV infection.

This study also found a striking difference in experience between those who accessed the home-based testing and the hospital-based testing. Interviewees expressed a high level of satisfaction with home-based testing in terms of their interactions with the service providers. Our findings also support the view that home-based testing effectively targets subpopulations that are less likely to be tested otherwise. Globally, HBTC in many resource-limited settings appear to be acceptable and have been shown to reach as high as two-thirds of the population that did not know their HIV status [21, 30, 31]. The experiences of interviewees with hospital-based testing on the other hand were predominantly negative. Provision of HIV testing services at healthcare facilities can be challenging. Healthcare providers faced with high workloads [32–34] and the general atmosphere at the hospital setting that requires healthcare providers to spend shorter times with patients in order to provide services to a large number creates a setting that is perceived as off-putting by older patients. Our results suggests that provision of HIV testing services in a home setting (instead of hospital-based testing) would be cost effective [35] and would make an impact in finding older adults who do not have varied opportunities to test or may avoid hospital-based testing due to the unsupportive environment and/or fear of ageist discrimination [36].

Although this study observed an unsupportive environment in hospital settings compared to that of the home setting, participants who tested in hospital settings were more likely to accept the diagnosis. Unlike patients in the hospital, who were usually experiencing a range of HIV-related symptoms, those tested in home settings were likely to be asymptomatic at the time of testing. They were less likely to accept their positive result. This might indicate that pre-testing counseling in the community setting is insufficient, possibly due to ageist beliefs both by healthcare providers and older adults themselves. Beliefs by healthcare providers that older adults are no longer sexually active and are not at risk of HIV infection [27, 37, 38] may lead to presumptions of negative HIV test results when providing testing services for older adults. Additionally, older adults’ beliefs that HIV is a disease of the ‘young’ [28, 38] may make them less likely to comprehend or ask questions during the pre-testing and counseling sessions. This study, therefore, cannot overemphasize the need for training of healthcare workers in offering counseling and testing services to older adults. HIV prevention messaging and awareness targeted towards older persons will also be key in changing older adults’ attitudes towards HIV.

In provision of HIV care, patient-centered care is key to achieving better health outcomes for the HIV-infected [39, 40]. Older adults in our setting would prefer service providers of the same gender and a similar age because they felt that older healthcare providers would be more understanding of their needs. However, in Kenyan public care setting providing HIV services, patients are not able to choose healthcare providers, and there are currently no efforts made to employ particular service providers to cater to the needs of older people. Our results support studies from other settings that have shown that older people are likely to feel comfortable and discuss sexual matters with care provider of the same gender [41]. HIV care programming at facility level and possibly community level should be structured to focus on these needs of older people seeking HIV testing and care services.

While this study has highlighted potential areas for improvement in providing HIV testing services to older persons, the study had some limitations. We interviewed older adults who were still engaged in HIV care services and did not include persons who were lost to follow-up. Experiences of those who were lost to follow-up may likely be different from those of participants we interviewed. In addition, our study did not interview the healthcare providers who would shed light on their interactions with older adults. Despite this, we believe that these results are important in informing HIV care programming for HIV testing service provision in an attempt to meet Kenya’s testing targets of 80% and ultimately the UNAIDS 90–90-90 targets [42].

## Conclusion

This study explored HIV testing experiences among HIV infected older adults. HIV testing services in health care facilities are not tailored to target older adults in our setting resulting in late diagnosis among older persons. Community level testing services, including home-based counseling and testing, on the other hand, provide adequate testing and counselling time which is key for older persons understanding of HIV positive results. Finding HIV infected older persons by targeting testing services to them is a step in attaining the first 90 of the UNAIDS targets of having 90% of HIV infected getting to know their HIV status.

## Additional files

Additional file 1: In-depth interview guide (PDF 191 kb)

Additional file 2: Focus group discussion guide (PDF 185 kb)

## Abbreviations

AMPATH: Academic Model Providing Access to Healthcare
ART: Antiretroviral treatment
DTC: Diagnostic testing and counseling
FGD: Focus group discussion
HBCT: Home-based counseling and testing
HIV: Human immunodeficiency virus
IDI: In depth interview
MTRH: Moi Teaching and Referral Hospital
PITC: Provider Initiated testing and counseling
UNAIDS: Joint United Nations Programme on HIV/AIDS
WHO: World Health Organization
