# Supplementary material for: HIV testing and counselling experiences: a qualitative study of older adults living with HIV in western Kenya
Source: BMC Geriatr. 2018 Oct 25;18:257. doi: 10.1186/s12877-018-0941-x (PMC6203278; doi:10.1186/s12877-018-0941-x)
Supplement: Supplementary file 1 — In-depth interview guide (PDF 191 kb) [file 12877_2018_941_MOESM1_ESM.pdf]

In-Depth Interview guide

Study Title: Characteristics, outcomes and experiences of HIV infected adults aged 50 years and older in western Kenya

Semi-Structured Interview Questions for Understanding the Experiences and healthcare needs of older adults living with HIV in western Kenya

Interview date: ___/___/___ (Date/Month/Year)

Start time: __________________

Introduction: Research assistant to introduce him/herself. Explain study procedures and details to participant, including audio recording, confidentiality, and rights to refuse participation. Obtain written informed consent. If accompanied, request the participant if they have someone they wish to join the interview with.

1. Begin by taking participants' socio-demographic characteristics
    a. Age (in years)? Get from file Age at first testing (years)
    b. Sex: Male or Female?
    c. Marital status: Single, Married, Divorced, Separated, Widowed, Re-married
    d. Highest formal educational level: None, Primary, Secondary, Tertiary
    e. Ask if they are staying alone or with family member – If possible get specifics of who he/she is staying with.

2. Let us begin by discussing about HIV, the general knowledge on HIV.
    a. Please tell me what you know about HIV? How is it transmitted? How does one prevent him/herself from getting infected? *Probe for when they got to know about this information (Was it before or after their HIV positive diagnosis)*

3. Please think about the time before you were tested for HIV, can you tell me about that time?

4. Let us now discuss about the time you were tested for HIV, please tell me what happened that led to you testing?
    a. Could you tell me about the HIV testing process?
    b. What was going on in your mind at that time?
    c. Did you suspect you could be HIV infected?
        i. Why? Why not?
    d. Could you please tell me about the time after the testing? What did you do? How did you feel?

5. Let us now focus on your life after knowing your status and now living with HIV?
   Probe for
    a. Have you been able to tell anyone about your status? Please describe to me how the process was? What was the person's reaction? Have you told anyone else?

b. How would you describe your relationship with (family members, colleagues/work mates, church members, community)? Has your relationship changed since you got to know about your status?
c. Please describe to me any support that you have access to? Probe for how he/she gets the daily food? Money for daily care as well as transport to the health facility for clinic visits? If a member of any support groups, social network, religious group?
d. *For participant on ART*: Please describe to me your experience with ART medication. Any other medication being taken along with ART? If on multiple medication, how they identify which one to take at what time? Any time they have missed medication? (*What was happening around the times they missed?)* Any side effects? (*Probe for when this occurred, did they continue with medication, did they inform the healthcare worker?)*
For participant not on ART: Do you know why you have not been started on ART? Did you start and stop? (*What happened?*) Are you on any other medication? (*For which other conditions?*)

6. Let us now discuss about the hospital where you are receiving care. Could you describe to me your experiences seeking care at the facility whenever you visit?
   a. Please describe your experience in keeping to your routine healthcare appointments
   b. How would you describe the communication with healthcare providers?
   c. What is satisfying about the HIV care services at the clinic?
   d. What is frustrating about the HIV care services at the clinic?
   e. How do healthcare providers treat you? Is it different from those younger than you?
   f. People living with HIV experiences a number of challenges. For example sometimes patients forget to take their medication or even forget to use condoms during sex. How easy is it for you to talk to your clinician about some of the challenges you face?
      i. Are you free to talk to your clinician about anything? What about when you have not adhered to your medication? How about discussions on sexual activity?
   g. Do you have some specific needs that you feel are not being met at the healthcare facility? Or by healthcare providers? *Probe on how those needs could be met from their perspective.*

7. Would you please share with me something that you would love to that we have not discussed?

<u>Closing</u>: Thank the participant for their time and information provided. Assure the participant about the privacy and confidentiality of the interview and the information. Ask if they have any questions about anything that was discussed. Ask if there is anything that wasn't raised that should have been that is important to know in understanding HIV care among the older adults. Provide reimbursement to the participant. Provide <u>researcher's contact</u> information for any future questions they may have.

*End time*___________________________
