# Supplementary material for: HIV testing and counselling experiences: a qualitative study of older adults living with HIV in western Kenya
Source: BMC Geriatr. 2018 Oct 25;18:257. doi: 10.1186/s12877-018-0941-x (PMC6203278; doi:10.1186/s12877-018-0941-x)
Supplement: Supplementary file 2 — Focus group discussion guide (PDF 185 kb) [file 12877_2018_941_MOESM2_ESM.pdf]

## Focus Group Discussion Guide

**Study Title:** Characteristics, outcomes and experiences of HIV infected adults aged 50 years and older in western Kenya

Semi-Structured focus group discussion guiding questions to understand the Experiences and healthcare needs of older adults living with HIV in western Kenya.

Interview date: ___/___/___ (Date/Month/Year)

Start time: __________________

Venue: ____________________

Introduction: Facilitator to introduce him/herself together with the note-taker. Explain study procedures and details to participants, including audio recording, confidentiality, and rights to refuse participation. Set the participation norms including one person speaking at a time, and that there are no right or wrong answers. Discuss if phone should be switched off or be on silent mode. Obtain verbal consent before commencing the FGD. Ask if there is need for any clarification before starting

1. Begin with participant's introduction with their ages taking participants' socio-demographic characteristics
    a. Age (in years)?
    b. Marital status: Single, Married, Divorced, Separated, Widowed, Re-married
    c. Highest formal educational level: None, Primary, Secondary, Tertiary

2. Begin by discussing about HIV, the general knowledge on HIV.
    *a.* Please tell me what you know about HIV? How is it transmitted? How does one prevent him/herself from getting infected? *Probe for when they got to know about this information (Was it before or after their HIV positive diagnosis)*

3. Let us now discuss about the time of HIV testing. What happened that led to your testing?
    a. How was the HIV testing process?
    b. What did the person testing you tell you?
    c. Did you suspect you could be HIV infected? Why? Why not?
    d. How about the time after the testing? What happened? What did the person testing you do? What about you? What did you do? How did you feel?

4. Let us now focus on life after knowing your HIV status and now living with HIV?
    a. What are some of the challenges you face as an older person living with HIV? Probe for adherence to medication and clinic visits, medication toxicities, other conditions suffered other than HIV, social support, stigma and discrimination by family, friends, community?

5. Let us now discuss about the hospital where you are receiving care. What has been your experience seeking care at the facility whenever you visit? What is satisfying about the healthcare services you receive at the facility? What is frustrating?

Probe for their communication with healthcare providers, quality of care facilities, time taken with providers, timing of the scheduled visits, working hours at the care facility, access (navigation) to the facility, age and gender of healthcare providers, queues at the clinics, care for comorbidities.

6. Is there anyone else who like to share something that we have not discussed?

Closing: Thank the participants for their time and information provided. Assure the participant about the privacy and confidentiality of the interview and the information. Ask if they have any questions about anything that was discussed. Ask if there is anything that wasn't raised that should have been that is important to know in understanding HIV care among the older adults. Offer refreshments and provide reimbursement to the participant. Provide researcher's contact information for any future questions they may have.

*End time*__________________________
